# T2T-YAO Reference Genome of Han Chinese — New Step in Advancing Precision Medicine in China

**DOI:** 10.1016/j.gpb.2023.09.001

**Published:** 2023-09-22

**Authors:** Xue Zhang

**Affiliations:** McKusick-Zhang Center for Genetic Medicine, State Key Laboratory of Medical Molecular Biology, Institute of Basic Medical Sciences, Chinese Academy of Medical Sciences & Peking Union Medical College (CAMS&PUMC), Beijing 100005, China

One of the many aims of precision medicine is to tailor disease prevention, including diagnostics and treatment, to everyone’s genetic makeup. Its foundation is built upon a set of linear chromosome sequences with outstanding accuracy, and it is such accuracy that assures the maximal anchorage of genetic and physical markers for disease mapping and molecular studies. In addition to the quality of a reference genome, its relevance to a real population is crucial; T2T-YAO represents one of the aboriginal Han Chinese populations born along the Yellow River basin [1].

The first step of human genomics can be traced back to the identification of the diploid 46 chromosomes of human genome by a Chinese named Tjio JH and his colleague Levan A in 1956 [2], [3], [4]. Building population-centric, complete, and accurate reference genomes is the fundamental task of genomics, and has been one of the long-standing goals since the initiation of the Human Genome Project some three decades ago. Chinese scientists have not only made significant contributions to this great project [5] and its several smaller-scale sequels but also to many subsequent genome-wide association studies (GWAS) for various human diseases.

It is noteworthy that the reference genome is dynamic and needs to be continually updated to reflect the diversity of populations worldwide. The completion of genome assemblies of Chinese individuals, such as YH [6], HX1 [7], NH1.0 [8], HJ [9], Han1 [10], and CN1 [11], has enabled precision medicine based on the most comprehensive and accurate genetic information. Recently, T2T-CHM13, published in April 2022 by the Telomere-to-Telomere Consortium, has assembled a complete and accurate haploid human genome for the first time, which is undoubtedly a remarkable achievement in this field [12].

Despite progresses made in sequencing technologies and assembly algorithms, T2T-CHM13 is based on a hydatidiform mole cell line with nearly homozygous genome instead of a real diploid human genome, to simplify and reduce the difficulties in assembly. Therefore, due to the limitations of sequencing technology, constructing a high-quality T2T diploid genome of a real individual remains challenging even after the completion of T2T-CHM13. Now, Gao and Kang’s groups have jointly assembled a diploid human genome called TAT-YAO from real individuals, which possesses a high quality comparable to CHM13 with no artificial sequences or model nucleotides and less than one error per ∼ 29 Mb.

Pangenomic studies have revealed more differences among ethnic groups and subpopulations than previously thought after sorting out the most repetitive regions. Both pangenomic studies and T2T-YAO, the population-centric reference genome assembly, demonstrate urgent needs for more population-stratified reference genomes. T2T-YAO is sampled from aboriginal human settlements at the starting point of the Hongtong migration, which represents a high-quality genome for Han Chinese subpopulation with clear ancestry markers and potentially can be used to trace back early settlements in this region. T2T-YAO provides an overview of the genetic distance between the Chinese and European populations, which includes ∼ 300 Mb (10% genome) exclusive sequences and ∼ 3000 unique genes to Chinese, as well as numerous structural variations. Such distance reflects genetic hallmarks of the Chinese population and is important for future functional annotations of these useful genome variables.

T2T-YAO, currently the highest-quality diploid genome published, marks a new start for a collection of a series of population-centric reference genomes for the Han Chinese populations and subpopulations. The completion of T2T-YAO represents a significant advancement in genome sequencing and assembly technology, enabling investigations into the allelic impacts on phenotype in a diploid setting. It is essential for the implementation of precision medicine and high-accuracy population genomics in China. Population genomics is a field of genetics that focuses on studying genetic variations within a population using a reference genome as sustainable coordinates. By comparing personalized genetic variations in a population or a subpopulation, researchers are able to identify allelic frequencies and distributions of genomic variants to understand the dynamics of population divergence, selection, and migration, as well as how genetic variations contribute to population adaptation, evolution, and health. A high-quality reference genome, such as T2T-YAO, will facilitate genetic studies with commonly-used methods for identifying genetic variants associated with specific phenotypes or diseases, such as GWAS analysis.

With the accumulation of related databases and knowledge about genetic variations that contribute to disease susceptibility and treatment response, precision medicine would become practicable. Another key requirement for precision medicine is a high-quality reference genome that can be used as a template for identifying variants. The genetic data of a given patient can be obtained through genetic sequencing or other methods, then, the raw data needs to be processed, analyzed, and interpreted to guide personalized medical treatment. This process usually focuses on genetic variations relevant to the patient's phenotypes and comparing them with a reference genome to determine their impacts on disease risk and treatment response. Based on genetic data and information obtained from the comparison with the reference genome, healthcare providers can tailor personalized treatments according to an individual’s genetic makeup and specific disease risks. Additionally, treatment plans can be adjusted based on continuously updated genetic information, and such a precise reference certainly provides sustainable coordinates for monitoring genetic and genomic details in diagnostics and therapeutics.

Although precision medicine has not yet been widely applied and is still a developing field, the potential benefits of precision medicine are enormous. T2T-YAO is just a new beginning of Chinese population genomics in China, more and further investigations are on their way. With continuous advances in genetic testing and analysis, this field will keep expanding, making it feasible to provide personalized treatment options for more patients in the future.

## Competing interests

The author declares no competing interests.

## CRediT authorship contribution statement

**Xue Zhang:** Conceptualization, Writing – original draft, Writing – review & editing. The author has read and approved the final manuscript.
